# Time trends in incidence and mortality of respiratory diseases of high public health relevance in Germany

**DOI:** 10.17886/RKI-GBE-2017-061

**Published:** 2017-10-09

**Authors:** Henriette Steppuhn, Silke Buda, Antje Wienecke, Klaus Kraywinkel, Kristin Tolksdorf, Jörg Haberland, Detlef Laußmann, Christa Scheidt-Nave

**Affiliations:** Robert Koch Institute, Department of Epidemiology and Health Monitoring, Berlin

**Keywords:** RESPIRATORY DISEASES, LUNG DISEASES, INCIDENCE, MORTALITY, SURVEILLANCE

## Abstract

Respiratory diseases are major causes of disease burden and mortality throughout the world. In Germany, alongside acute respiratory infections (ARI), chronic lung diseases – including lung cancer, chronic obstructive pulmonary disease (COPD), and asthma – are of particular socioeconomic importance. ARI incidence rates differ significantly according to age, season and year. They are recorded as weekly consultation rates as reported by selected outpatient and inpatient care facilities. Between 2009 and 2016, the highest incidence rates of severe acute respiratory infection (SARI) were recorded among young children in outpatient (9.4%) and inpatient (0.2%) care. Mortality rates for ARI are also subject to seasonal and annual fluctuations. However, the official statistics on causes of death, which lead to estimates of more than 17,000 annual deaths, provide an inadequate measure of death rates because chronic underlying illnesses are often recorded as the cause of death rather than a more recently acquired acute infection. Therefore, the excess mortality caused by ARI needs to be assessed in the context of influenza outbreaks. Regarding lung cancer, COPD and asthma, the long-term time trends in disease incidence and mortality rates are of particular interest from a health policy perspective. Analyses of data from the official statistics on causes of death for the years 1998 through 2015 show that mortality rates for lung cancer and COPD decreased on average by 1.8% and 1.1% per year respectively, among men, whereas among women they increased by 2.5% (lung cancer) and 2.3% (COPD) annually. Nevertheless, more men than women died of lung cancer or COPD in 2015 in Germany: 29,378 men and 15,881 women died from lung cancer, and 17,300 men and 13,773 women died from COPD. During the same period, the asthma mortality rates decreased on average by 8.3% annually among women and by 11.2% annually among men, and the absolute number of deaths came down to 659 among women and 393 among men. Lung cancer incidence rates have been at similar levels as lung cancer death rates since 1998. No such data are available on time trends in COPD or asthma incidence rates. Coordinated surveillance of respiratory diseases needs to be expanded within the framework of international action plans for disease prevention.

## 1. Introduction

In Germany and throughout the world, diseases of the airways and lung are of significant socioeconomic importance [1-7]. Within the current framework of the international system of disease classification (10th revision of the International Statistical Classification of Diseases and Related Health Problems, ICD-10), this group of diseases not only covers a wide range of acute and chronic disorders of the respiratory system (J00-J99), but also malignant neoplasms (cancer) of the trachea, bronchus and lung (C33-C34) [2]. Recent data from Germany (2015) show that 12.3% of all deaths are caused by respiratory diseases (ICD-10: J00-J99 including C33-C34) [8]. The direct costs incurred from the treatment of respiratory disorders alone (including C33-C34) amount to EUR 14.7 billion (2008) [2]. Respiratory system disorders – alongside musculoskeletal disorders and connective tissue diseases – were the most common reason that patients sought treatment in a doctor’s practice in 2016 [9]. They are also among the most important factors that cause people to take time off work [2, 10]. In Germany, alongside acute diseases of the respiratory tract (J00-J22), lung cancer (C33-C34), chronic obstructive pulmonary disease (COPD, J44), and asthma (J45-J46), in particular, are associated with a high burden of disease and mortality [1-7, 11, 12]. They are, therefore, of particular relevance to public health and are also the central focus of the World Health Organization’s (WHO) surveillance and prevention strategies [1, 11-13].

Lung cancer (C33-C34) and COPD (J44) among both genders, and pneumonia (pneumonia, organism unspecified J18) among men, count among the ten most frequent causes of death in Germany [8]. The importance of these diseases is also reflected by the impact on the health care system. Every year, seasonal influenza is a major cause of extra visits in ambulatory care [14]. Depending on the season, influenza can cause up to 30,000 hospitalisations due to complications of primary influenza and secondary bacterial infection [14]. The number of pulmonary infections that occur outside of hospital (community-acquired pneumonia) is estimated at between 400,000 and 600,000. Approximately 30% to 50% of these cases lead to hospitalisation [15, 16]; community-acquired pneumonia should be treated in accordance with the S3 guideline [17]. Nevertheless, pneumonia is often the result of hospital-acquired (nosocomial) infections. According to a study of the burden of disease associated with nosocomial infections in Europe, the incidence of nosocomial pneumonia (healthcare-associated pneumonia – HAP) is 138 per 100,000 population [18]. Calculated in DALYs (disability-adjusted life years), which is an internationally established standard for measuring the burden of disease caused by an illness or pathogen, HAP (with a rate of 169 per 100,000 individuals) is the most important disease in terms of severity and case fatality among the six frequently occurring nosocomial infections examined in this study. However, only data from hospitals were taken into account in this case – data from nursing homes were not included [18]. In addition, COPD and asthma are not only common causes of outpatient care visits [19]. As chronic conditions that can be treated in an ambulatory care setting, both diseases are among the major causes of potentially avoidable hospital admissions with more than 270,000 hospitalisations in 2015 [20-23]. Around 190,000 cases of lung cancer were treated in hospitals in 2015, which provides an illustration of the considerable level of medical care associated with this disease [23, 24].

**Info box 1: Measures of disease frequency**

*Prevalence*: The prevalence is the frequency of a certain disease within a defined population in a specific period of time. It is usually expressed as the percentage of the people who have had a certain disease, e.g., within the last 12 months (12-month prevalence) or during their life (lifetime prevalence).

*Incidence*: The incidence is the frequency of new cases of a certain disease within a defined population in a specific period of time. It is often expressed as a percentage of new cases within a specific period (cumulative incidence) or as the number of new cases per 1,000 person-years (incidence rate).

*Cumulative incidence:* The number of new cases is divided by the original number of people at risk. In other words, this is the proportion of a population that could develop the disease in a defined period of time (for example, at the beginning of a twelve-year study period). In order to calculate the cumulative incidence of a certain disease, new cases are counted only among people who did not have the disease at the start of the study (Annex Table 3).

*Incidence rate:* The incidence rate is the number of new cases divided by the so-called person-time at risk. The person-time at risk corresponds to the duration of time during which the people in a defined population are at risk of developing a disease. It can vary from person to person. For example, not everyone is at risk of developing a certain disease for the entire study duration because they may develop the disease before the study ends (Annex Table 3) [41]. According to international standards for cancer registration, the annual incidence rate of lung cancer is calculated as the proportion of the average total population of the respective reference year that develops lung cancer in that year [24]. The number of people with previously diagnosed lung cancer among the total population is considered negligible. Thus, the average total population of the respective reference year is simplified and equated with the number of people at risk for that year. The incidence rate is presented as the number of new cases of lung cancer per 100,000 population.

Source: [24, 41, 129]

Continuous and timely disease monitoring including incidence and mortality rates is indispensable in infection control and also forms the basis of the WHO’s Global Action Plan for the Prevention and Control of NCDs 2013-2020 [12, 13]. At the Robert Koch Institute (RKI), assessments of the rates of new cases and deaths (incidence and mortality, see Info box 1, 2, 3 and 4) associated with acute respiratory infections (ARI) and cancer, including lung cancer, are carried out continuously as part of the ongoing surveillance of infectious diseases and the pooled analyses of federal state-level epidemiological cancer registry data held by the German Centre for Cancer Registry Data (ZfKD). In addition, data on the epidemiology of respiratory diseases are available from the population-based, representative health surveys carried out at regular intervals by the RKI and from the official statistics on causes of death published by the Federal Statistical Office (Info box 3). All these data are integrated into the German Federal Health Monitoring information system [25]. Additional information on the prevalence (Info box 1) of lung cancer is published at regular intervals by the ZfKD [24]. Two Fact sheets published in this issue use data from the German Health Update (GEDA 2014/2015-EHIS) nationally health interview survey to provide current assessments of the prevalence of known COPD and asthma among adults. The current analyses are based on data available at the national level to describe time trends in the incidence and mortality due to acute respiratory infections and chronic lung diseases of high relevance to public health.

## 2. Methods

The current investigation relies on data sources available at the national level to analyse time trends in incidence (Info boxes 1 and 2) and mortality rates (Info boxes 3 and 4) of acute respiratory infections (ARI), lung cancer, COPD and asthma. It includes data on ARI from primary care practices and hospitals (syndromic surveillance), [26, 27] from the RKI’s Centre for Cancer Registry Data (ZfKD) [28, 29], and from the official statistics on causes of death [8] (Info boxes 3, 5, 6 and 7). In addition, the incidence rate of asthma among adults during the observation period between 1997-1999 and 2008-2011 was estimated based on data from the nationwide interview and examination surveys regularly conducted by the RKI as part of the German Federal Health Monitoring system [30-33] (Info box 8).

### 2.1 Acute respiratory infection (ARI)

#### Case definition

In primary care practices, an acute respiratory infection (ARI) was counted if a doctor diagnosed a disease by entering at least one of the following ICD-10 codes into the medical information system: J00-J22 (J00-J06: acute upper respiratory infections; J09-J18: influenza and pneumonia; J20-J22: other acute lower respiratory infections) or an individual diagnosis of J44.0 (chronic obstructive pulmonary disease with acute lower respiratory infection) or B34.9 (viral infection, unspecified). In order to assess the burden of severe acute respiratory infections (SARI) in inpatient care, cases with a discharge diagnosis of influenza, pneumonia or other acute lower respiratory infection (ICD-10 J09-J22, Table 1) were selected.

#### Incidence of acute respiratory infection (ARI)

A syndrome is a typical combination of several symptoms or diagnoses (or, in this case, the ICD-10 codes used to describe them) associated with a particular disease. The RKI conducts syndromic surveillance of acute respiratory infection (ARI) in both outpatient (general practitioners and paediatricians) and inpatient (hospital) care. It is particularly used to monitor the conditions resulting from influenza, as influenza viruses can cause acute respiratory diseases, and unlike many viruses causing the common cold, can account for serious illnesses and death. The RKI’s Working Group Influenza (Arbeitsgemeinschaft Influenza, AGI) has provided data on outpatient care for many years (see Info box 5). Moreover, an ICD-10-code based, electronic reporting system was included in the syndromic routine surveillance since 2012/2013. This includes keeping electronic records of diagnostic codes [26]. The weekly ARI consultation rate (see Info box 2) is an important indicator for ARI activity. Due to its strong association with age, the consultation rate is estimated in five age groups separately [27]. For severe acute respiratory infections (SARI), the RKI has also developed a continuous syndromic sentinel surveillance system in hospitals (the ICOSARI project) within a scientific cooperation with the HELIOS Kliniken GmbH (see Info box 6). An important indicator in this regard is the weekly SARI hospitalisation rate (see Info box 2). Just as the ARI consultation rate, these estimates are also reported stratified by five age groups (0-4, 5-14, 15-34, 35-59 and 60 years of age or older) [14, 34].

**Info box 2: The consultation and hospitalisation incidence**

*The consultation incidence of acute respiratory infection (ARI):*

Visits to a doctor because of a medical condition in order to gain medical advice (and treatment) can also be called a consultation. The ARI consultation incidence refers to the number of people per 100,000 population who have visited (consulted) a general or paediatric practice due to a new acute respiratory infection in a given week. If the weekly ARI consultation incidence is assessed by age groups, the values are based on 100,000 population of the respective age group. Doing so demonstrates that children aged 4 years or below have the highest risk of developing a medically attended ARI. This indicator can also be expressed as a percentage (as a proportion of 100 population), resulting in a weekly ARI rate.

*The hospitalisation incidence of severe acute respiratory infection (SARI):*

If someone is treated for an acute respiratory infection in hospital, it can be termed that he or she was hospitalised due to the infection in question. The SARI hospitalisation incidence is the number of people per 100,000 population who have been hospitalised due to a severe acute respiratory infection (SARI) in a given week. If the weekly SARI hospitalisation incidence is assessed by age groups, the values are based on 100,000 population from the respective age group. Doing so demonstrates that children aged 4 years or below and elderly people aged 60 years or above have a higher risk of being treated in a hospital for severe respiratory infections than children older than 4 years of age or adults below the age of 60 years. This indicator can also be expressed as a percentage (as a proportion of 100 population), resulting in a weekly SARI rate.

Source: [27]

#### Mortality of severe acute respiratory infections (SARI)

Age and gender-specific mortality rates for severe acute respiratory infections are based on data from the official statistics on causes of death provided by the Federal Statistical Office (Info box 3 and 4). Trends in mortality rates for severe acute respiratory infections were examined for the years 1998 through 2015 (see 2.5).

### 2.2 Lung cancer

#### Case definition

**Annex Figure 1:**
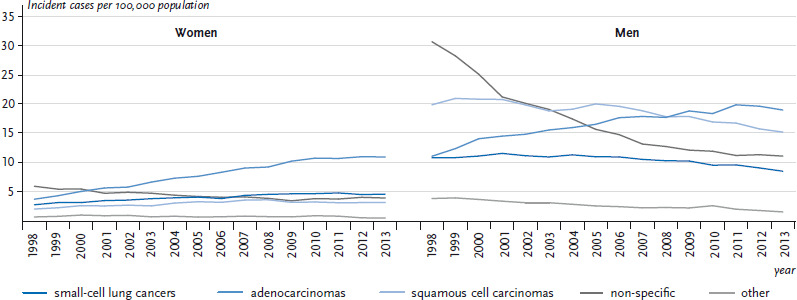
**Trends in age-standardised incidence rates (incident cases per 100,000 population) for malignant neoplasms of the bronchus and lung (ICD-10 C34) between 1998 and 2013, Germany (selected registries; old European standard population; ≥0 years of age) by gender and type of lung cancer histology:** In accordance with the current International Classification of Diseases for Oncology (ICD-O-3), the figures were divided into small-cell lung cancers (8041-8045), adenocarcinomas (8140-8384), squamous cell carcinomas (8050-8080), non-specific lung carcinomas (8000-8005), and other malignant lung carcinomas (8010-8011) Data Source: German Centre for Cancer Registry Data [24, 28-29]

The definition of incident cases and deaths from lung cancer was made in accordance with the current international classification of diseases (10th revision of the International Statistical Classification of Diseases and Related Health Problems, ICD-10) and is based on the following ICD-10 codes: C33 for very rare malignant neoplasms of the trachea, and C34 for malignant neoplasms of the bronchus and lung (Table 1). In order to differentiate among the different types of lung cancer, the morphology codes recorded in the cancer registries were classified as follows (in accordance with the current International Classification of Diseases for Oncology – ICD-O-3): small cell lung carcinomas (8041-8045), adenocarcinomas (8140-8384), squamous cell carcinomas (8050-8080), unspecified lung tumours (8000-8005), and other malignant lung carcinomas (8010-8011) (Annex Figure 1).

#### Incidence of lung cancer

The analyses of lung cancer incidence rates are based on data from the epidemiological cancer registries of the federal states, which transmit data annually to the German Centre for Cancer Registry Data (ZfKD) in accordance with the Federal Cancer Registry Data Act (Info box 7). The nationwide incidence of lung cancer (Info box 1) was calculated using this data [29]. Trends in lung cancer incidence rates were examined for the years 1998 through 2013 (see 2.5).

#### Mortality of lung cancer

Age and gender-specific mortality rates for lung cancer are based on data from the official statistics on causes of death provided by the Federal Statistical Office (Info box 3 and 4). Trends in lung cancer mortality rates were examined for the years 1998 through 2015 (see 2.5).

### 2.3 Chronic obstructive pulmonary disease (COPD)

#### Case definition

The case definition of deaths from COPD was based on the ICD-10 code (Info box 3) J44 for other chronic obstructive pulmonary disease [2] (Table 1). In order to ensure comparability with previous analyses of COPD mortality, ICD-10 codes for COPD and other chronic lower respiratory diseases (ICD-10: J40-J44, J47) were considered in combination; only the ICD-10 codes for asthma (J45-J46) were excluded (Table 1) [35-37].

**Info box 3: Causes of death**

*Causes of death:* The official statistics on causes of death provide information about the most important causes of death and trends in death rates. From 1 January 1998 onwards, the 10th revision of the International Statistical Classification of Diseases and Related Health Problems (ICD-10) has been in use to code the causes of death that occurred during the observation period. The statistics on causes of death are derived from an evaluation of death certificates. These are issued individually on the basis of the federal states’ burial laws for all deaths as well as for stillborns with a birth weight of at least 500g and are pooled at the Federal Statistical Office.

Source: [129]

#### Mortality of COPD

Age and gender-specific mortality rates for COPD are based on data from the official statistics on causes of death provided by the Federal Statistical Office (Info box 3 and 4). Trends in COPD mortality rates were examined for the years 1998 through 2015 (see 2.5).

### 2.4 Asthma

#### Case definition

Based on data from the national health interview and examination surveys, incident cases of asthma (Info box 8) were defined as those participants who reported to have ever been diagnosed with asthma by a physician and having had asthma and/or having used asthma medication in the past 12 months. The case definition of death from asthma was based on the asthma-specific ICD-10 codes (Info box 3) J45-J46 [2] (Table 1).

#### Incidence of asthma among adults

The current analysis is based on data from the German Health Interview and Examination Survey for Adults (DEGS1) conducted by the RKI as part of the continuous health monitoring system in Germany [30-33]. DEGS1 has a mixed study design which allows for both cross-sectional and longitudinal analyses [30-32]. The study’s concept and design is described in detail elsewhere [30-32]. Gender-specific estimates of the cumulative incidence of asthma (Info box 1) were calculated based on data from 3,959 participants of the DEGS1 study who participated in both the baseline survey (GNHIES98, 1997-1999) and the follow-up survey (DEGS1, 2008-2011) (see Info box 8). Participants (n=290) were excluded from the analyses because they reported to have ever been diagnosed with asthma by a physician at the time of the baseline survey (1997-1999) or because complete information about their asthma status was unavailable at baseline and/or follow-up survey. All results were reported by gender and further stratified by two age groups (18-44 and 45-79 years of age) (Table 4). Estimates on asthma incidence were calculated by using a specific weighting factor in order to correct not only for deviations of the baseline sample from the population structure at the time of data collection, but also to adjust for selective participation at follow-up [38]. For comparability with other studies, asthma incidence rates per 1,000 person-years were also estimated (Info box 1). Due to varying degrees of disease activity over time in individuals with asthma and resulting effects on self-reported information from those that are affected, different selection criteria of study participants for analyses on asthma incidence (population at risk, Info box 1) are found in the literature [39-42]. Therefore, incidence rates were additionally calculated after excluding specific participants who may have already had asthma before the baseline survey was conducted (see Annex Table 3).

#### Mortality of asthma

Age and gender-specific mortality rates for asthma are based on data from the official statistics on causes of death provided by the Federal Statistical Office (Info box 3 and 4). Trends in asthma mortality rates were examined for the years 1998 through 2015 (see 2.5).

**Info box 4: Mortality**

*Mortality:* Mortality is the frequency of deaths within a given population during a specific period of time. It is usually expressed as a percentage of deaths within a certain period (cumulative mortality) or as the number of deaths per 1,000 person-years (mortality rate).

*Cause-specific mortality rate:* The cause-specific mortality rate is based on the annual number of deaths attributed to a specific underlying cause as recorded in the official statistics on causes of death (Info box 3). The average total population of the respective reference year is considered as the number of people at risk. Therefore, the annual number of deaths, for example, from lung cancer, is divided by the average total population of the respective reference year and is presented as the number of deaths from lung cancer per 100,000 population.

Source: [24, 129]

### 2.5 Analysis of time trends

The Joinpoint Regression Program (version 4.4.0.0, [43]) was used to analyse time trends in incidence and mortality rates per 100,000 population. The average annual percentage change (AAPC) in the age-standardised rates (Info box 9) at the national level was estimated by performing joinpoint regression analyses [43, 44]. Trends in the incidence rates for lung cancer were examined for the years 1998 through 2013, whereas trends in mortality rates for each included respiratory disease were examined for the years 1998 through 2015. In each analysis, years (‘joinpoints’) were identified, in which statistically significant changes in trends occurred. Such joinpoints indicate, for example, points at which incidence rates begin increasing more noticeably or level out [44]. In order to account for the impact of changes to the population age structure, the rates were standardised according to age using the old European standard population (Info box 9). In addition, time trends in mortality rates for asthma and COPD were compared to time trends in mortality rates for all causes [37, 45]. For all outcomes of interest, the number of absolute cases as well as the number of cases per 100,000 population were presented by calendar year. Population estimates reflect the average total population of the respective reference year. For the years 1998 through 2010, these figures were based on projections of census data collected in the former FRG in 1987 and in the former GDR in 1990 [8]. From 2011 onwards, projections obtained from the 2011 census were used [8]. All results were reported by gender (see Tables 2 and 3) and further stratified by age groups (0 to 54, 55 to 74, and 75 years of age or older, see Annex Tables 1 and 2). In contrast to the other age-associated chronic respiratory diseases considered here, asthma occurs frequently among people of all ages and may overlap with a COPD from mid- to late adulthood [2, 24, 46-50]. For international comparability, time trends in asthma mortality rates were thus additionally analysed for people aged 5 to 34 years [51].

## 3. Results

### 3.1 Acute respiratory infections

**Figure 1:**
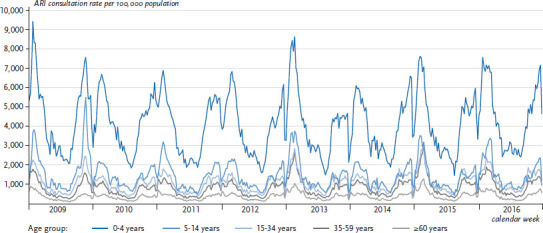
Trends in the incidence of acute respiratory infections (ARI, ICD10: J00-J22, J44.0, B34.9) based on the weekly consultation rates (per 100,000 population) between 2009 and 2016, Germany (men and women) by age Data source: Syndromic surveillance of acute respiratory infections (ARI) by the AGI (the RKI’s working group on influenza) [26, 27]

Figure 1 shows the weekly changes in the consultation incidence of acute respiratory infections that occurred between 2009 and 2016. Depending on the week and season, up to 2.6% of the population (at the peak of the 2012/2013 influenza season) visited a general or paediatric practice due to an acute respiratory infection. Large differences between age groups were identified year-round, whereas gender-specific differences hardly played a role (data not shown). Each week, between 1.5% and 9.4% of children aged 0 to 4 years visited a paediatrician. By contrast, no more than 1.3% of people aged 60 years or older visited their general practitioner due to an acute respiratory complaint – even in winter during weeks with high levels of influenza activity.

Only a small proportion of acute respiratory infections is severe enough to result in hospital treatment. Hospital admission due to severe acute respiratory infection (SARI) is also highly dependent on age. The weekly incidence of SARI (hospitalisation incidence, Info box 1) is highest among children aged 0 to 4 years. In the period of 2009 to 2016, it ranged from 0.001% to 0.19% depending on the season and level of influenza activity. Between 0.01% and 0.06% of adults aged 60 years or older were admitted to a hospital each week. The risk of a serious illness increases with age and with the presence of other risk factors, such as chronic pre-existing illnesses.

**Figure 2:**
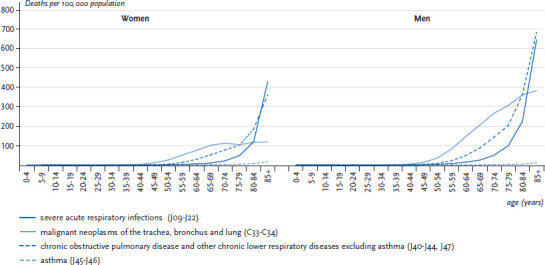
Age-specific mortality rates (deaths per 100,000 population) due to respiratory diseases of high public health relevance in 2015, Germany (≥0 years of age) by age and gender Data source: Official statistics on causes of death [8]

**Figure 3:**
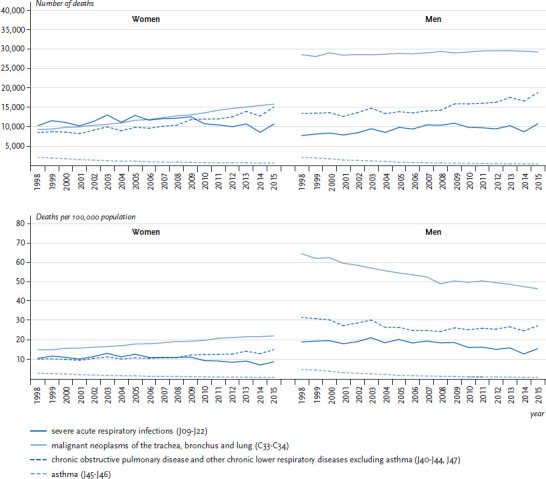
Trends in the annual number of deaths (above) and age-standardised mortality rates (deaths per 100,000 population, below) due to respiratory diseases of high public health relevance between 1998 and 2015, Germany (old European standard population; ≥0 years of age) by gender Data source: Official statistics on causes of death [8]

In 2015, a severe acute respiratory infection (ICD-10: J09-J22) was reported as the underlying cause of death (monocausal cause of death) for 10,743 women and 10,816 men in Germany (Table 1). The crude mortality rate was 25.9 per 100,000 among women, which is very similar to the rate found among men (26.9). However, mortality rates increased significantly with age for both genders (Figure 2). Between 1998 and 2015, age-standardised mortality rates due to severe acute respiratory infections significantly declined on average (AAPC) by 1.8% annually among women and by 1.9% annually among men (Figure 3, Table 2). Most recently, a similar annual drop in mortality rates (APC, from 2005 onwards) of 4.3% among men and 3.9% among women (see Table 2) has been observed. The overall decrease in mortality due to severe acute respiratory infection is mainly attributable to a marked decline among elderly (≥75 years) women and men (Annex Table 1).

### 3.2 Lung cancer

**Figure 4:**
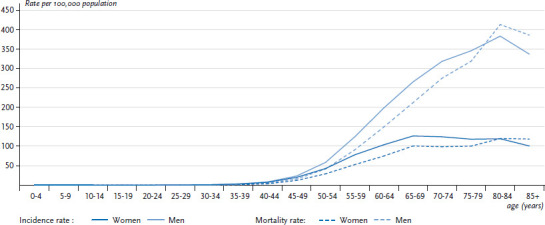
Age-specific incidence and mortality rates (per 100,000 population) for malignant neoplasms of the trachea, bronchus and lung (ICD-10 C33-C34) in 2013, Germany (≥0 years of age) by gender Data Sources: German Centre for Cancer Registry Data [28, 29] and Official statistics on causes of death [8]

**Figure 5:**
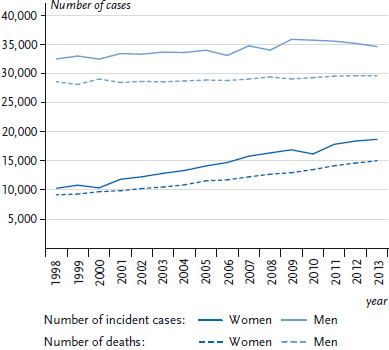
Trends in the annual number of incident cases and deaths, malignant neoplasms of the trachea, bronchus and lung (ICD-10 C33-C34) between 1998 and 2013, Germany (≥0 years of age) by gender Data Sources: German Centre for Cancer Registry Data [28, 29] and Official statistics on causes of death [8]

**Figure 6:**
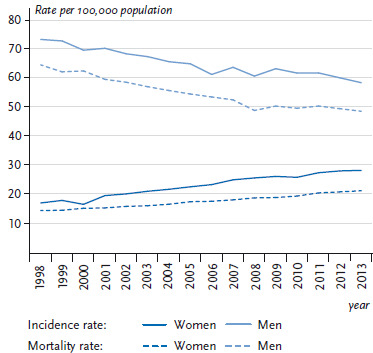
Trends in age-standardised incidence and mortality rates (per 100,000 population) for malignant neoplasms of the trachea, bronchus and lung (ICD-10 C33-C34) between 1998 and 2013, Germany (old European standard population; ≥0 years of age) by gender Data Sources: German Centre for Cancer Registry Data [28, 29] and Official statistics on causes of death [8]

18,810 women and 34,693 men in Germany were diagnosed with lung cancer (ICD-10: C33-C34) in 2013 (see Figure 5). The average age at diagnosis was around 69 years. The crude incidence rate was substantially higher among men (87.9 per 100,000) than among women (45.7 per 100,000) (data not shown) and increased with age particularly among men (Figure 4). The absolute number of cases increased since 1998 among women but remained relatively constant among men. Correspondingly, sex-specific differences were found in the time trends of age-standardised incidence rates (Figure 6). Whereas among men the lung cancer incidence rate decreased on average (AAPC) by 1.5% annually between 1998 and 2013, women showed a contrasting trend with an average annual increase of 3.3% (Table 3). However, among women under 55 years of age, a 1.4% annual drop in the incidence of lung cancer can be observed from 2007 onwards (Annex Table 2). When lung cancer incidence among women is examined according to morphological type, a larger increase can be identified for adenocarcinomas compared to squamous cell carcinomas and small cell carcinomas. Adenocarcinomas also appear to be increasing among men despite the overall trend towards decreasing lung cancer incidence rates. However, it is important to recognise that the incidence rate of tumours classified as ‘unspecified’ also decreased during this period (Annex Figure 1) [24].

In 2015, lung cancer was reported as the underlying cause of death among more men (29,378) than women (15,881) (Table 1). The average age at death from lung cancer was 71 years. The crude mortality rate of 73.1 per 100,000 was considerably higher among men than among women (38.3 per 100,000) and substantially increased with age particularly among men (Figure 2). In a manner similar to the incidence, there has been a clear increase since 1998 in the absolute number of deaths among women, whereas deaths among men remained largely unchanged during the same period (Figure 3; Figure 5). Differences were also found between age-standardised death rates among women and men (Figure 3; Figure 6). Among men, there was a significant overall average annual decline of 1.8% (Table 2). Through 2006, this reduction was somewhat more marked (2.3%) before levelling out at 1.4% thereafter. The decrease in lung cancer mortality rates from 2007 onwards is more noticeable among younger men (<55 years old) compared to older men (≥55 years old) (Annex Table 1). Among women there was, by contrast, a significant increase in lung cancer mortality of 2.5% annually over the entire observation period. However, as with the incidence, a reduction in lung cancer mortality of 2.8% per year was observed among younger women (<55 years old).

### 3.3 COPD

In 2015, COPD (ICD-10: J44) was reported as the underlying cause of death among more men (17,300) than women (13,773) (Table 1). The crude death rate was substantially higher among men (43.1 per 100,000 population) than among women (33.2 per 100,000 population) and strongly increased with age particularly among men. Similar results were also seen when ICD-10 codes of COPD and other chronic lower respiratory diseases excluding asthma were considered (ICD-10: J40-J44, J47) (Table 1, Figure 2). This combination of ICD-10 codes is used internationally to enable comparability of long-term trends in COPD mortality over time, and is therefore also used below (see 2.3).

Figure 3 shows that the absolute number of deaths in 1998 was considerably lower among both women and men than in 2015. However, time trends in age-standardised death rates differed between women and men (Figure 3 and Table 2). Among women, there was a significant increase of 2.3% annually over the entire observation period (Table 2). At the same time, an average annual increase in COPD mortality rates was most noticeable among women aged 55 to 74 years (Annex Table 1). Among men, there was, by contrast, a significant overall average decline (AAPC) in COPD mortality rates of 1.1% annually (Table 2). However, this decrease came to a halt in 2007. A continuous decrease in mortality rates was only seen among men aged 75 years or older (Annex Table 1). Gender differences can also be observed when time trends in COPD mortality rates are compared to time trends in all-cause mortality (Table 2): between 1998 and 2015, a similar average decline in COPD and in all-cause mortality rates was observed among men (-1.1% and -1.8% annually). By contrast, time trends in COPD and all-cause mortality differed among women: COPD mortality rates rose by 2.3% annually as opposed to a significant decrease in all-cause mortality rates of 1.4% annually.

### 3.4 Asthma

During an average observation period of approximately 12 years, the cumulative incidence (Info box 1) of asthma was higher among women (4.1%) than among men (1.6%) (Table 4). This gender difference was observed both at a younger age (18 to 44 years) and among older adults (45 to 79 years). To enable comparability with other studies, incidence rates were also calculated (Info box 1). Depending on the criteria considered for the selection of study participants in the analyses, asthma incidence rates ranged from 2.7 to 3.4 per 1,000 person-years among women and from 1.1 to 1.3 per 1,000 person-years among men (Annex Table 3).

**Info box 5: Data from the syndromic surveillance of acute respiratory infection (ARI) by the Working Group Influenza (Arbeitsgemeinschaft Influenza, AGI)**

**Data holder:** Robert Koch Institute

**Aims:** Providing timely year-round surveillance data of acute respiratory infections (ARI) in primary care with a focus on influenza activity. This includes weekly reporting of the surveillance results.

**Method used to estimate consultation incidence:** Weekly number of cases with medically attended acute respiratory infection (ARI) in sentinel practices of RKI’s Working Group Influenza

**Population:** Total population that sought a consultation with a general practitioner or paediatrician

**Participants:** Approximately 700 primary care physicians that regularly report to the RKI

**Coverage:** The participating sentinel practices provide medical care to about 1% of the total population

**Study period:** Cases identified between 1 Jan 2009 and 31 Dec 2016 have been included in the results presented here

Source: [26, 27]

More information in German is available at

https://influenza.rki.de/Default.aspx

Among women and men, asthma (ICD-10: J45-J46) was rarely documented as the underlying cause of death in 2015 (Table 1). The crude asthma mortality rate was similar in women (1.6 per 100,000 population) and men (1.0 per 100,000 population) and increased with age among both genders (Figure 2). Figure 3 shows that the number of deaths from asthma among women and men in 1998 was considerably higher than in 2015. Between 1998 and 2015, there was a strong decline in age-standardised asthma mortality rates, which was even more pronounced among men than among women (AAPC:-11.2% vs. -8.3%; Table 2). An annual decline in asthma mortality rates of 6.8% among women and of 7.5% among men was also observed in the age group 5 to 34 years (data not shown). Finally, among both genders, a much faster average annual decline in asthma mortality rates than in all-cause mortality rates was observed in all age groups (AAPC for women aged ≥0 years: -8.3% vs. -1.4%; AAPC for men aged ≥0 years: -11.2% vs. -1.8%; Table 2).

## 4. Discussion

### 4.1 Acute respiratory infection

Syndromic surveillance of acute respiratory infections with a focus on influenza is an internationally recognised and established practice [52]. Laboratory analyses are only conducted in a very small proportion of cases with an acute respiratory infection. Moreover, 75% of all patients who were treated in inpatient care for community acquired pneumonia left hospital with a diagnosis of J18 (pneumonia, unspecified organism) [53]. As such, assessing the individual diagnoses of influenza (instead of the fixed diagnostic groups used in syndromic surveillance) would probably provide an incomplete and distorted depiction of the situation. Nevertheless, the syndromic approach has a clear disadvantage: the burden of disease caused by individual pathogens such as influenza can only be estimated using relatively complex statistical procedures, which, at least in some cases, need to be accompanied by representative microbiological examinations [12, 54]. Influenza infections and subsequent pneumonia can also occur in hospitals. Such nosocomial pneumonia is often caused by pathogens that are already found in the respiratory tract (‘commensals’) which are normally harmless and only cause disease once the mucous membrane of the respiratory tract has been weakened, for example, due to an influenza infection [55].

The occurrence of acute respiratory infections is characterised by strong seasonal fluctuations. As such, a close (weekly) assessment of consultation rates is as relevant to health policy as analyses of long-term trends. In general, acute respiratory illnesses during the winter are significantly more pronounced in both outpatient and inpatient care than during the summer months. Although efficient health care systems, such as the one in Germany, are designed with this in mind, seasonal influenza waves occur in various strengths. A particularly severe wave of influenza can tax the resources of outpatient and inpatient care for a few weeks. Furthermore, medical staff can also be affected by these outbreaks and may fall ill during periods that already require greater level of medical care. Finally, people with chronic diseases are at a higher risk during these periods, as an additional respiratory infection could lead to a severe or even fatal disease; this also increases the level of care needed during influenza outbreaks.

**Info box 6: Data from syndromic surveillance of inpatient cases of severe acute respiratory infections (ICO-SARI)**

**Data holder:** Robert Koch Institute

**Aims:** Providing reliable and up-to-date information on serious acute respiratory infections (SARI), with the possibility of comparing different seasons and corresponding data from other countries.

**Method used to estimate hospitalisation incidence:** Weekly data on the number of patients with a main discharge diagnosis of SARI from one of the hospitals participating in the ICOSARI project (sentinel hospitals)

**Population:** The population in the catchment area of the sentinel hospitals, with the possibility of extrapolation to the total population in Germany

**Participants:** Sentinel hospitals belonging to HELIOS Kliniken GmbH (83 in 2014)

**Coverage:** In 2014, the participating sentinel hospitals in 13 out of 16 federal states provided care to about 6% of all hospital patients across Germany

**Study period:** Cases identified between 1 Jan 2009 and 31 Dec 2016 have been included in the results presented here Source: [14, 34]

Even if respiratory infections contribute to or actually cause death, they are rarely recorded as the monocausal cause of death; instead, an underlying condition is usually documented in the statistics on causes of death. Therefore, mortality rates and absolute number of deaths due to severe acute respiratory infections are likely underestimated, despite the fact that the mortality rate for community acquired, hospital-treated pneumonia is already high (at approximately 13% depending on age) [53]. As such, it is an international standard practice to estimate the influenza-related deaths by utilising the total number of deaths by any cause (Info box 10). This method usually involves calculating a background mortality rate – the all-cause mortality rate that would have been expected at the time of the influenza season if there had been no influenza virus circulation. If the total mortality rate observed during the influenza season is markedly higher than the background mortality, the difference between expected and observed mortality rates can be attributed to the disease. This is referred to as excess mortality and can be estimated using statistical methods [56]. In the case of severe influenza seasons, such as those that occurred in 2012/2013 and 2014/2015, an excess mortality of 26 per 100,000 has been identified [14, 57]. This corresponds to 20,700 and 21,300 influenza-related deaths during the respective periods. Clearly, only a fraction of influenza-related deaths are stated as such in the annual statistics on causes of death due to severe respiratory infections. However, many respiratory infections that occur during an influenza season – including the majority of pneumonia cases – are not caused by influenza; this applies even more to respiratory-coded deaths that occurred outside the influenza season.

### 4.2 Lung cancer

In the case of lung cancer, continuous and nationwide estimates of incidence and mortality rates are made in accordance with the Federal Cancer Registry Data Act of 2009. Consistent with developments in the incidence rates, age-standardised mortality rates of lung cancer have been declining for decades among men and are still rising substantially among women. Due to the poor prognosis associated with lung cancer (about half of patients die within one year of being diagnosed) [24], the trends in mortality reflect the trends in incidence.

As the majority of lung cancer in Germany can be attributed to smoking, the different temporal trends found among women and men can be traced to changes in smoking habits that occurred towards the end of the 20th century. Whereas the proportion of smokers decreased among men, it rose among women until about 2000, although it never reached the same level as among men [58]. Women have now also begun reducing their tobacco consumption, and this will have a significant effect in the coming decades. The rate of new lung cancer cases among women below the age of 55 years has fallen by 1.4% per year since 2007, and the mortality rate in this age group has been decreasing by 2.8% per year since 2008. This trend has been observed in almost all Western industrialised countries. In fact, in the US and some Scandinavian countries, a reduction in lung cancer incidence and mortality has already been observed among women overall, although the peak rates in these countries were substantially higher than in Germany. There are clear regional differences in lung cancer mortality within the EU, and this is particularly the case among men: rates in some Eastern European countries are approximately three to four times higher than, for example, in Finland or Sweden [24].

**Info box 7: Data from the German Centre for Cancer Registry Data (ZfKD)**

**Data holder:** German Centre for Cancer Registry Data at the Robert Koch Institute; epidemiological cancer registries of the German federal states

**Aims:** Aggregating data from the epidemiological registries of the federal states in order to estimate the nationwide number of new cancer cases as well as prevalence and survival rates in Germany, stratified by cancer diagnosis, age and gender.

**Registration method:** Registration of all new cancer cases using electronic or paper reports filed by the doctors who diagnosed the case

**Population:** The population with residency in Germany (all age groups)

**Coverage:** Since 2009, complete nationwide coverage; the year in which registration began varies by state and ranges from 1970 (Saarland) to 2009 (Baden-Württemberg). Currently, a registration completeness rate of 90-95% (nationwide) can be assumed for all types of cancer

**Study period:** Cases diagnosed between 1 Jan 1998 and 31 Dec 2013 have been included in the results presented here

Source: [28, 29]

More information in German is available at

www.krebsdaten.de

Passive smoking, radon gas, other forms of environmental pollution (especially particulates) and exposure to asbestos can also increase the risk of lung cancer. Long-term exposure not only to particulates themselves [59] but also to the pollutants that they carry increases the likelihood of lung cancer [60, 61]. Whereas tobacco consumption is the major risk factor of lung cancer in Europe and North America, exposure to air pollution from open fires used for cooking and heating within households is an additional cause of lung cancer in large parts of sub-Saharan Africa and in some regions of Asia and Oceania [62]. The increase in the proportion of adenocarcinomas observed in Germany has also been observed in many other countries over the last few decades [63, 64]. Compared to the other two main forms of lung cancer (squamous cell carcinoma and small cell lung carcinoma), adenocarcinoma is less strongly associated with smoking and is the most common form of lung cancer in non-smokers [65]. In this respect, a growing influence of environmental risk factors, such as particulate air pollution, which are especially associated with adenocarcinomas, could play a role. However, the evidence in this regard is still limited [66], and particulate matter pollution (measured as PM10 values) seems to have declined in Germany since the mid-1990s, even if the values recommended by the WHO are still frequently exceeded in urban areas [67]. Other possible explanations for the increase in adenocarcinomas could be changes in the levels of tobacco consumption, the composition of tobacco products, the depth of inhalation and the age at which people begin to smoke.

### 4.3 COPD

Consistent with results on lung cancer mortality, in 2015, COPD was recorded as the underlying cause of death among more men than women. However, due to different time trends in COPD mortality between women and men a further narrowing of the gender gap in death rates for COPD can be observed as already reported in Germany and other European countries since 1994 [35]. As with lung cancer, smoking is the most important modifiable risk factor of COPD in Germany [48, 68]. The risk of developing COPD depends on the total amount of cigarette smoking over time (‘pack years’) [2, 7]. However, reliable data on time trends in COPD morbidity or survival rates are not available in Germany [2, 48, 69]. Since a high proportion of people with unknown or undiagnosed COPD within the population is to be expected, this would require a consideration of reliable data based on a pulmonary function test (spirometry) [70-73]. In the absence of such data, it cannot be determined to what extend differing time trends in COPD mortality between women and men can be attributed to gender differences in the development of COPD incidence – e.g., due to secular trends in smoking – or survival rates and resulting changes in prevalence (Info box 1) over time [74, 75]. In addition, COPD is a long-term chronic condition that may progress over decades accompanied by increasing health-related disability [2, 50]. Assessments of the burden of disease associated with COPD therefore highly depend on the availability of reliable data permitting analyses on time trends in the number of years spent with health-related disability [4, 5, 7]. In turn, this requires a reliable database for the analysis of trends in the prevalence of COPD and COPD-related disability over time [4, 5, 7].

**Info box 8: Data from the panel component of the German Health Interview and Examination Survey for Adults (DEGS)**

**Data holder:** Robert Koch Institute

**Aims:** Providing information on the incidence and course of major diseases and associated risk factors between 1997-1999 and 2008-2011

**Methods:** Paper-and-pencil self-administered questionnaire, physical examinations and laboratory tests, a physician-administered computer-assisted medical history taking, computer-assisted medication review

**Population:** People (aged 18 to 79 years at baseline) with permanent residency in Germany

**Sampling:** Stratified two-stage sample in which adults from 120 municipalities in Germany were randomly selected from population registries

**Participants:** 7,124 adults (18-79 years) for the baseline survey; 3,959 adults (28-91 years) for the follow-up

**Response rate:** 61% at baseline; 62% at follow-up

**Study period:** Baseline survey in 1997-1999 (GNHIES98); follow-up survey in 2008-2011 (DEGS1)

Source: [30-32]

More Information in German is available at

www.degs-studie.de

In international comparison, such as with data from Hannover (2006), there is a regional association between the local average amount of cigarette smoking over the time (‘pack years’) and the prevalence of COPD [76]. In Spain, moreover, a decline in COPD prevalence estimates based on spirometry testing was observed among adults aged 40 to 69 years between 1997 and 2007. This decline in prevalence was recorded across all age groups among men and in particular among women under 50 years of age [77]. The authors discussed that in addition to changes in smoking habits, prenatal and early-childhood influences may be contributing to COPD morbidity in later life [77]. Impaired growth and functional development of the lungs also relates to COPD risk [50, 78, 79]. In addition to a genetic predisposition and prenatal influences (e.g., maternal smoking during pregnancy), repeated respiratory infections in early childhood, exposure to airborne pollutants, or childhood asthma may contribute to developing COPD in later adulthood [48, 50, 68, 78-80]. Further work-related factors (such as exposure to coal dust) are also important modifiable risk factors of COPD [48, 68, 81]. Moreover, exposure to high levels of air pollution in households that, for example, result from burning biomass (such as wood or animal dung) in open fires or leaky stoves is also a known cause of COPD [2, 3, 50, 82, 83]. This is particularly the case in low and middle income countries [3, 82, 83]. The survival prospects of people with COPD are further influenced by the quality of medical care, including prevention and treatment of concurrent conditions and sequelae (such as cardiovascular disease, but also respiratory infections and lung cancer) [50].

### 4.4 Asthma

Consistent with results observed at the global level, age-standardised asthma mortality rates and annual number of asthma deaths have both substantially decreased among women and men in Germany [51, 84]. The considerable faster decline in asthma mortality when compared with all-cause mortality indicates that survival prospects of asthma patients may have improved. This development has been related particularly to improvements in drug therapy of asthma patients [51, 84]. However, low asthma mortality rates could probably be reduced even further, particular through the implementation of appropriate medical care, including adequate management of the condition by patients themselves [51, 85]. In the current analyses, the incidence rate of asthma during the observation period between 1997-1999 and 2008-2011 among women was higher than among men. Results from other epidemiological studies also point to an increased asthma risk for women compared to men, whereby this difference is already evident in adolescence, and besides hormonal factors, differences in lung development or in the vulnerability (susceptibility) to environmental factors have been related to the observed gender differences in asthma incidence [42, 86-90]. Further, no nationwide studies are available on long-term trends in asthma incidence or survival rates in Germany [2, 91, 92].

**Info box 9: Age-standardised incidence and mortality rates**

*Age standardisation*: Age standardisation is used to compare measures of disease frequency or mortality rates (Info box 1 and 2) of populations with different age structures. It is used when investigating trends within a geographically defined population to account for age structure changes over time. To calculate an age standardisation, ‘crude’ incidence or mortality rates for individual age groups are determined and applied to a standard population with a fixed age distribution. In the current analyses of time trends in incidence and mortality rates, the ‘old’ European standard population is used as the standard population. This statistical method enables analyses of incidence and mortality rates that are independent of changes and differences in age structure of the population.

*Age-standardised incidence and mortality rates*: This can be illustrated with the example of lung cancer among men (Figure 5 and 6). In 2013 in Germany, 34,693 incident cases of lung cancer (ICD-10: C33-C34) were recorded within the framework of nationwide cancer registration – more than the 32,589 that were recorded fifteen years earlier in 1998. Does this mean that men now more frequently develop lung cancer? This question can be examined using the age-standardised incidence rate: the age-standardised incidence rate for lung cancer in 1998 was 73.4 per 100,000 population, whereas in 2013 it was 58.6 – a substantially lower rate. When age-standardisation is employed to take the ageing population into account, it becomes clear that the rate of incident cases of lung cancer among men has in fact decreased considerably during the period in question.

The average annual percentage change (AAPC) was -1.5%, which was statistically significant. Therefore, the rate of incident cases of lung cancer has actually decreased by 1.5% per year. The absolute number of incident cases rose because the number of people in older age groups increased. The observed time trends in the annual number of incident cases and age-standardised incidence rates for lung cancer among men were comparable to the corresponding time trends in the annual number of deaths and age-standardised mortality rates. Age standardisation enables evaluation of time trends in incidence and mortality rates, preventing an increasing number of older people within a given population from influencing the results.

Source: [129]

Repeated population-based surveys have, however, demonstrated that the prevalence (Info box 1) of asthma increased during the second half of the last century not only in Germany, but also in other parts of the world [46, 93-102]. This increase has been linked to changes in lifestyle and environmental factors (such as infections, exposure to microbes, allergens and airborne pollutants, or diet) possibly resulting in an increment in asthma incidence particularly among children and young adults [46, 94-96, 99, 103]. Most recently, a further rise in the lifetime prevalence of physician-diagnosed asthma among both genders was recorded from national interview and examination surveys among adults (1997-1999 and 2008-2011) as well as among children and adolescents (2003-2006 and 2009-2012) [47, 49, 104]. At the same time, an increase in the 12-month prevalence was also observed, which, among adults, was attributable mainly to an increase in prevalence among women [49, 105]. In addition to changes in asthma incidence, improved survival of asthma patients could have contributed to the observed changes in prevalence. In large population-based epidemiological studies, data on asthma prevalence and incidence is based on self-reported information and particularly relies on the assessment of self-reported physician-diagnosed asthma [46, 98, 106-111]. Thus, secular trends in degrees of asthma awareness or changes in the way patients perceive symptoms and seek medical care over time may all affect levels of self-reported asthma prevalence and incidence [94, 95, 106, 112].

### 4.5 Summary overview and conclusions

Both the burden of acute respiratory infections and the resulting need for medical care are subject to strong seasonal fluctuations. A close (weekly) surveillance of shortterm trends in incidence is thus as relevant to health policy as the analysis of long-term trends over time. A large proportion of influenza-related deaths are attributed to existing chronic underlying diseases or to complications frequently associated with bacteria-related pneumonia instead of the acute influenza infections themselves. Therefore, evaluations of mortality using the official statistics on causes of death are inadequate and additional assessments of the excess mortality associated with influenza are necessary. For that purpose, the method of estimating influenza-related excess mortality has been implemented in Germany (Info box 10) [56]. However, compared to other countries, timely data that could provide an up-to-date assessment of time trends in the influenza-attributable mortality in Germany are lacking [113].

Regarding chronic lung diseases including lung cancer, long-term trends in incidence and mortality rates are of particular relevance. A decline in asthma mortality rates is observed among both genders. However, mortality rates for COPD and lung cancer are decreasing among men, whereas they are increasing among women. Incidence trends for longer periods of time are only available for lung cancer and remain at a very similar level to mortality rates. The contrasting developments in lung cancer incidence and mortality among women and men can be attributed to sex differences in smoking habits that occurred in the second half of the 20th century – the proportion of men who smoked was already declining during this time, whereas it was still increasing among women [58]. Overall, the proportion of smokers in Germany has decreased in recent years, but still around one quarter of adults is currently smoking (women: 20.8%, men: 27.0%, see also fact sheet on Smoking among adults in Germany in issue 2/2017). The sustainable reduction of tobacco consumption is therefore an important goal in terms of preventing lung cancer and COPD as well as other chronic diseases. In addition, some of the burden of disease and premature mortality associated with acute respiratory infections could be avoided by vaccination (influenza and pneumococcal vaccination) [114, 115]. This is particularly the case among elderly patients with multiple chronic diseases, as they are significantly more likely to suffer from a severe illness which could even have a fatal outcome [114].

The results of the present investigation illustrate a number of aspects that are of particular relevance for the continuous analysis of time trends in morbidity and mortality of respiratory diseases. Detailed analyses of gender and age-specific trends enables conclusions to be drawn about changes in underlying behavioural or environmental determinants of disease that may explain these trends (for example, temporal changes in tobacco consumption and the incidence rates for lung cancer). At the same time, trends in morbidity and mortality for age-associated diseases such as COPD and lung cancer are also influenced by changes to the population age structure. This can be seen, for example, in the difference in time trends between the absolute number of incident cases and age-standardised incidence rates for lung cancer among men (Info box 9). Clearly, temporal changes in mortality rates can also be influenced by changes in survival, such as through improved treatments. Regarding lung cancer, survival rates are estimated regularly in Germany as part of national cancer surveillance activities in accordance with the Federal Cancer Registry Data Act [24, 116, 117]. For COPD and asthma, mortality follow-up of national health survey participants could help to close this gap in the future [74, 118, 119]. Additional information can be derived by considering process data of in- and outpatient care [75, 120].

Based on data from the official statistics on causes of death, the present analysis investigated time trends in mortality of severe acute respiratory infections and chronic lung diseases, including lung cancer with a high relevance to public health. Compared to lung cancer, which represents a clearly defined underlying disease, there is a far higher risk of misclassification for COPD or asthma as the underlying cause of death, not least because of the possible overlap of both diseases in middle and older age [50, 121-123]. However, a decline in asthma mortality was evident also among younger people aged 5 to 34 years. The fact that patients with COPD often suffer from other chronic diseases, such as cardiovascular disease or lung cancer, can also affect the risk of death [123, 124]. Furthermore, it is particularly difficult to identify the single cause of death in COPD patients with severe acute respiratory infections [123, 124]. Besides, the analysis of time trends in mortality based on data from the official statistics on causes of death faces a limitation due to the fact that coding practices can change over long periods of time. For example, since the death certificates are processed electronically in England and Spain using the IRIS software, dementia is now documented more frequently as an underlying cause of death instead of aspiration pneumonia (pneumonia due to substances entering the lungs, such as after swallowing food). In these cases, by contrast, manual coding would more likely have resulted in an acute respiratory illness being documented as the underlying cause of death [125, 126]. In Germany, the electronic coding of causes of death using IRIS software has been piloted at the regional level since 2007; some federal states now use the system to process all of their death certificates electronically [127]. Improving the statistics on causes of death through quality control in death registration and the implementation of multi-causal coding of the underlying diseases that led to the death represents a major challenge for the future [127, 128].

**Info box 10: Excess mortality of influenza [6]**

*Influenza-related excess mortality:* Excess mortality refers to an increased rate of death among a particular population compared to the population’s average rate, or an increased number of deaths over a certain period of time compared to the normally expected death rate at that time of year. In order to calculate influenza-related excess mortality, the background mortality rate is usually calculated – this is the mortality rate that would be expected during the respective period (month, week) if no influenza activity were present. If the level of mortality present during the influenza season is – to a greater or lesser extent – higher than the background level, this excess mortality can be attributed to influenza [6].

More information in German is available at http://www.rki.de/DE/Content/Infekt/EpidBull/Archiv/2015/Ausgaben/03_15.pdf

### 4.6 Conclusion

This article underlines the need for continuous and coordinated surveillance of morbidity and mortality related to acute respiratory infections as well as lung cancer, COPD and asthma as chronic lung diseases of high public health relevance. In doing so, it is essential that the association between respiratory diseases and the concurrence of other, mainly age-related, diseases is taken into account. The analysis of existing data – also with regard to their limitations and the potential for future development – is a particularly important step in establishing sustainable surveillance structures in Germany within the framework of international public health strategies aimed at the prevention and control of communicable and non-communicable diseases.

## Key statements

- The incidence rate of acute respiratory infections is highly dependent on age and fluctuates strongly according to the time of year and the severity of the influenza season.
- The mortality rate for severe acute respiratory infections is not recorded adequately by statistics on causes of death; therefore, additional estimates need to be made of the excess mortality associated with influenza outbreaks.
- Long-term time trends in the incidence and mortality of chronic lung diseases, including lung cancer, are of particular interest from a health policy perspective.
- Lung cancer incidence and mortality rates decreased among men and increased among women.
- COPD mortality rates decreased among men and increased among women, whereas asthma mortality rates substantially declined for both genders.

## Figures and Tables

**Table 1:** Mortality due to respiratory diseases including lung cancer in 2015, Germany (≥0 years of age) by gender Data source: Official statistics on causes of death [8]

ICD-10 codes		Gender	Number of deaths	Deaths per 100 thousand population (crude rate)
**J00-J99**	Diseases of the respiratory system	Women	31,700	76.4
		Men	36,600	91.1
**J09-J22**	Severe acute respiratory infections (SARI)^1^	Women	10,743	25.9
		Men	10,816	26.9
**J40-J44, J47**	COPD and other chronic lower respiratory diseases excluding asthma	Women	15,166	36.5
		Men	18,877	47.0
**J44**	Chronic obstructive pulmonary disease (COPD)	Women	13,773	33.2
		Men	17,300	43.1
**J45-J46**	Asthma	Women	659	1.6
		Men	393	1.0

**C33-C34**	Malignant neoplasms of the trachea, bronchus and lung	Women	15,881	38.3
		Men	29,378	73.1
**C34**	Malignant neoplasms of bronchus and lung	Women	15,870	38.2
		Men	29,354	73.1

^1^ Severe acute respiratory infections (SARI): influenza, pneumonia and other acute infections of the lower respiratory tract.

ICD-10=10^th^ revision of the International Statistical Classification of Diseases and Related Health Problems

**Table 2:** Trends in age-standardised mortality rates (deaths per 100,000 population) due to respiratory diseases of high public health relevance and for all causes of death between 1998 and 2015, Germany (old European standard population; ≥0 years of age) by gender Data source: Official statistics on causes of death [8]

ICD-10 codes		Gender	Period	APC	(95% CI)	Period	APC	(95% CI)	AAPC	(95% CI)
**J09-J22**	(Severe acute respiratory infections – SARI)	Women	1998-2005	1.9	-1.4/5.2	2005-2015	-4.3	-6.2/-2.4	-1.8	-3.4/-0.2
		Men	1998-2007	0.0	-1.9/1.9	2007-2015	-3.9	-5.9/-1.9	-1.9	-3.1/-0.6
**C33-C34**	(Malignant neoplasms of the trachea, bronchus and lung)	Women	1998-2015	2.5	2.4/2.6					
		Men	1998-2006	-2.3	-2.7/-2.0	2006-2015	-1.4	-1.7/-1.1	-1.8	-2.0/-1.6
**J40-J44, J47**	(COPD and other chronic lower respiratory diseases excluding asthma)	Women	1998-2015	2.3	1.8/2.9					
		Men	1998-2007	-2.7	-3.8/-1.5	2007-2015	0.8	-0.5/2.1	-1.1	-1.8/-0.3
**J45-J46**	(Asthma)	Women	1998-2006	-10.3	-11.2/-9.3	2006-2015	-6.5	-7.7/-5.4	-8.3	-9.0/-7.6
		Men	1998-2008	-13.7	-14.5/-12.9	2008-2015	-7.6	-9.9/-5.2	-11.2 -	12.2/-10.3
**A00-T98**	(All causes of death)	Women	1998-2015	-1.4	-1.6/-1.2					
		Men	1998-2008	-2.5	-2.9/-2.2	2008-2015	-0.8	-1.3/-0.2	-1.8	-2.1/-1.5

APC=annual percentage change, and 95% confidence interval (95% CI)

AAPC= average annual percentage change, and 95% confidence interval (95% CI) between 1998 and 2015

COPD=chronic obstructive pulmonary disease

**Table 3:** Trends in age-standardised incidence and mortality rates (per 100,000 population) for malignant neoplasms of the trachea, bronchus and lung (ICD-10 C33-C34) between 1998 and 2013, Germany (old European standard population; ≥0 years of age) by gender Data Source: German Centre for Cancer Registry Data [28, 29]

ICD-10 codes		Gender	Period	APC	(95% CI)	Period	APC	(95% CI)	AAPC	(95% CI)
**C33-C34**	(Malignant neoplasms of the trachea, bronchus and lung)	Women	1998-2008	3.9	3.7/4.2	2008-2013	2.1	1.4/2.7	3.3	3.1/3.6
		Men	1998-2005	-1.8	-2.1/-1.5	2005-2011	-0.8	-1.3/-0.3	-1.5	-1.9/-1.2
			2011-2013	-2.7	-4.8/-0.5					

APC=annual percentage change, and 95% confidence interval (95% CI)

AAPC=average annual percentage change, and 95% confidence interval (95% CI) between 1998 and 2013

**Table 4:** Cumulative incidence of asthma among adults (18-79 years of age) Data source: German Health Interview and Examination Survey for Adults 1997-1999 and 2008-2011 [30-32]

Gender	Age group	Cumulative incidence (95% CI)
**Women**	Total (18-79 years)	4.1 (2.8-5.7)
	18-44 years	3.6 (1.9-6.1)
	45-79 years	4.7 (2.8-7.3)
**Men**	Total (18-79 years)	1.6 (0.9-2.6)
	18-44 years	1.9 (1.0-3.5)
	45-79 years	1.2 (0.5-2.2)

CI=Confidence interval

## 5. Annex

**Annex Table 1:** Trends in age-standardised mortality rates (deaths per 100,000 population) due to respiratory diseases of high public health relevance and for all causes of death between 1998 and 2015, Germany (old European standard population; ≥0 years of age) by gender and age Data source: Official statistics on causes of death [8]

ICD-10 codes	Period	APC	(95% CI)	Period	APC	(95% CI)	AAPC	(95% CI)
**J09-J22 (Severe acute respiratory infections)**								
**Women**								
0-54 years	1998-2015	-1.0	-2.5/0.5					
55-74 years	1998-2015	-0.7	-1.6/0.2					
≥75 years	1998-2005	2.0	-1.3/5.5	2005-2015	-4.9	-6.8/-3.1	-2.1	-3.7/-0.5
**Men**								
0-54 years	1998-2015	-1.6	-2.4/-0.7					
55-74 years	1998-2015	-1.2	-1.9/-0.4					
≥75 years	1998-2005	0.8	-2.0/3.8	2005-2015	-3.7	-5.1/-2.2	-1.8	-3.2/-0.5
**C33-C34 (Malignant neoplasms of the trachea, bronchus and lung)**								
**Women**								
0-54 years	1998-2008	1.6	0.8/2.4	2008-2015	-2.8	-4.0/-1.6	-0.2	-0.8/0.4
55-74 years	1998-2003	2.7	1.4/4.0	2003-2015	3.9	3.6/4.2	3.6	3.2/3.9
≥75 years	1998-2015	1.6	1.5/1.8					
**Men**								
0-54 years	1998-2007	-2.6	-3.1/-2.0	2007-2015	-4.4	-5.2/-3.7	-3.5	-3.9/-3.0
55-74 years	1998-2007	-2.7	-3.0/-2.4	2007-2015	-0.8	-1.2/-0.4	-1.8	-2.1/-1.6
≥75 years	1998-2012	-1.0	-1.2/-0.7	2012-2015	-2.7	-5.1/-0.4	-1.3	-1.7/-0.9
**J40-J44, J47 (COPD and other chronic lower respiratory diseases excluding asthma)**								
**Women**								
0-54 years	1998-2015	1.5	0.6/2.5					
55-74 years	1998-2006	0.0	-1.7/1.6	2006-2015	6.4	5.2/7.7	3.3	2.4/4.3
≥75 years	1998-2015	1.5	1.0/2.0					
**Men**								
0-54 years	1998-2015	-0.6	-1.3/0.1					
55-74 years	1998-2008	-2.9	-4.1/-1.8	2008-2015	3.2	1.2/5.3	-0.4	-1.4/0.5
≥75 years	1998-2015	-1.4	-1.8/-1.0					
**J45-J46 (Asthma)**								
**Women**								
0-54 years	1998-2015	-7.5	-8.2/-6.8					
55-74 years	1998-2015	-9.6	-10.2/-8.9					
≥75 years	1998-2007	-10.4	-11.7/-9.1	2007-2015	-4.2	-6.3/-1.9	-7.5	-8.6/-6.4
**Men**								
0-54 years	1998-2008	-9.0	-10.7/-7.2	2008-2015	-2.7	-6.8/1.6	-6.4	-8.2/-4.6
55-74 years	1998-2008	-14.6	-15.6/-13.6	2008-2015	-8.1	-11.4/-4.6	-12.0	-13.3/-10.6
≥75 years	1998-2010	-14.1	-15.1/-13.1	2010-2015	-8.0	-14.1/-1.5	-12.3	-14.0/-10.6
**A00-T98 (All causes of death)**								
**Women**								
0-54 years	1998-2007	-2.5	-2.8/-2.2	2007-2015	-1.6	-2.0/-1.2	-2.1	-2.3/-1.8
55-74 years	1998-2007	-2.5	-2.8/-2.2	2007-2015	-0.2	-0.6/0.2	-1.4	-1.7/-1.2
≥75 years	1998-2015	-1.3	-1.5/-1.1					
**Men**								
0-54 years	1998-2003 2006-2015	-2.2 -2.3	-2.8/-1.6 -2.6/-2.1	2003-2006	-3.8	-6.5/-1.1	-2.6	-3.0/2.1
55-74 years	1998-2008	-2.9	-3.1/-2.6	2008-2015	-0.6	-1.1/-0.1	-1.9	-2.2/-1.7
≥75 years	1998-2008	-2.2	-2.7/-1.7	2008-2015	-0.4	-1.2/0.3	-1.5	-1.9/-1.1

APC=annual percentage change, and 95% confidence interval (95% CI)

AAPC=average annual percentage change, and 95% confidence interval (95% CI) between 1998 and 2015

COPD=chronic obstructive pulmonary disease

ICD-10=10^th^ revision of the International Statistical Classification of Diseases and Related Health Problems

**Annex Table 2:** Trends in age-standardised incidence rates (incident cases per 100,000 population) for malignant neoplasms of the trachea, bronchus and lung (ICD-10 C33-C34) between 1998 and 2013, Germany (old European standard population; ≥0 years of age) by age and gender Data Source: German Centre for Cancer Registry Data [28, 29]

ICD-10 codes	Period	APC	(95% CI)	Period	APC	(95% CI)	AAPC	(95% CI)
**C33-C34 (Malignant neoplasms of the trachea, bronchus and lung)**								
**Women**								
0-54 years	1998-2000	9.9	1.0/19.6	2000-2007	2.4	1.1/3.6	1.8	0.7/3.0
	2007-2013	-1.4	-2.5/-0.2					
55-74 years	1998-2005	4.2	3.9/4.5	2005-2008	5.7	3.8/7.6	4.3	4.0/4.7
	2008-2013	3.6	3.2/4.0					
≥75 years	1998-2004	2.2	1.4/2.9	2004-2008	4.5	2.5/6.5	2.1	1.5/2.7
	2008-2013	0.1	-0.7/0.9					
**Men**								
0-54 years	1998-2010	-2.0	-2.2/-1.8	2010-2013	-4.9	-6.4/-3.3	-2.6	-2.9/-2.3
55-74 years	1998-2000	-0.4	-2.9/2.2	2000-2005	-2.4	-3.2/-1.6	-1.3	-1.7/-0.9
	2005-2013	-0.8	-1.1/-0.5					
≥75 years	1998-2002	-2.3	-3.6/-1.0	2002-2011	-0.2	-0.6/0.2	-1.4	-2.0/-0.9
	2011-2013	-5.2	-8.5/-1.9					

APC=annual percentage change, and 95% confidence interval (95% CI)

AAPC=average annual percentage change, and 95% confidence interval (95% CI) between 1998 and 2013

**Annex Table 3:** Cumulative incidence and incidence rates (per 1,000 person-years, PY) for asthma among adults (18-79 years of age) Data source: German Health Interview and Examination Survey for Adults 1997-1999 and 2008-2011 [30-32]

	Cumulative incidence (95% CI)	Incidence rate per 1,000 PY (95% CI)	Incidence rate* per 1,000 PY (95% CI)
**Women**			
Total (18-79 years)	4.1 (2.8-5.7)	3.4 (2.2-4.6)	2.7 (1.7-3.6)
18-44 years	3.6 (1.9-6.1)	3.0 (1.3-4.6)	1.8 (0.8-2.8)
45-79 years	4.7 (2.8-7.3)	4.0 (2.2-5.8)	3.7 (2.0-5.5)
**Men**			
Total (18-79 years)	1.6 (0.9-2.6)	1.3 (0.7-2.0)	1.1 (0.5-1.7)
18-44 years	1.9 (1.0-3.5)	1.6 (0.7-2.6)	1.4 (0.5-2.3)
45-79 years	1.2 (0.5-2.2)	1.0 (0.3-1.6)	0.6 (0.2-1.0)

* in addition, participants of the follow-up survey in 2008-2011 (n=18) reporting a year of asthma diagnosis ≥5 years before the baseline survey (1997-1999) were excluded [41]

CI=Confidence intervall

PY=person years
